# Reduced Axon Calibre in the Associative Striatum of the *Sapap3* Knockout Mouse

**DOI:** 10.3390/brainsci11101353

**Published:** 2021-10-14

**Authors:** Eliana Lousada, Mathieu Boudreau, Julien Cohen-Adad, Brahim Nait Oumesmar, Eric Burguière, Christiane Schreiweis

**Affiliations:** 1 Team ‘Neurophysiology of Repetitive Behaviours’ (NERB), Institut du Cerveau, Inserm U1127, Centre National de la Recherche Scientifique (CNRS) U7225, Sorbonne Universités, Hôpital de la Pitié-Salpêtrière, 75013 Paris, France; eliana.lousada@icm-institute.org (E.L.); eric.burguiere@icm-institute.org (E.B.); 2 Montreal Heart Institute, Montréal, QC H1T 1C8, Canada; emb6150@gmail.com; 3 NeuroPoly Lab, Institute of Biomedical Engineering, Polytechnique Montréal, Montréal, QC H3T 1J4, Canada; jcohen@polymtl.ca; 4 Functional Neuroimaging Unit, Centre de Recherche de l’Institut Universitaire de Gériatrie de Montréal (CRIUGM), Université de Montréal, Montréal, QC H3W 1W5, Canada; 5 Mila—Quebec AI Institute, Montréal, QC H2S 3H1, Canada; 6 Team ‘Myelin Plasticity and Regeneration’, Institut du Cerveau, Inserm U1127, Centre National de la Recherche Scientifique (CNRS) U7225, Sorbonne Universités, Hôpital de la Pitié-Salpêtrière, 75013 Paris, France; brahim.naitoumesmar@icm-institute.org

**Keywords:** repetitive behaviours, structural cortico-striatal connectivity, *Sapap3* knockout mouse model, axon calibre, myelination, AxonDeepSeg, associative and sensorimotor striatum, compulsive-like and tic-like behaviours, obsessive–compulsive disorder, Tourette syndrome

## Abstract

Pathological repetitive behaviours are a common feature of various neuropsychiatric disorders, including compulsions in obsessive–compulsive disorder or tics in Gilles de la Tourette syndrome. Clinical research suggests that compulsive-like symptoms are related to associative cortico-striatal dysfunctions, and tic-like symptoms to sensorimotor cortico-striatal dysfunctions. The *Sapap3* knockout mouse (*Sapap3*-KO), the current reference model to study such repetitive behaviours, presents both associative as well as sensorimotor cortico-striatal dysfunctions. Previous findings point to deficits in both macro-, as well as micro-circuitry, both of which can be affected by neuronal structural changes. However, to date, structural connectivity has not been analysed. Hence, in the present study, we conducted a comprehensive structural characterisation of both associative and sensorimotor striatum as well as major cortical areas connecting onto these regions. Besides a thorough immunofluorescence study on oligodendrocytes, we applied AxonDeepSeg, an open source software, to automatically segment and characterise myelin thickness and axon area. We found that axon calibre, the main contributor to changes in conduction speed, is specifically reduced in the associative striatum of the *Sapap3*-KO mouse; myelination per se seems unaffected in associative and sensorimotor cortico-striatal circuits.

## 1. Introduction

Repetitive, ritualistic actions are a core feature of animal behaviour, including humans, which allow for efficient learning and automatisation of behaviour [1,2,3]. However, several neuropsychiatric and neurodevelopmental disorders, amongst them Gilles de la Tourette syndrome (TS), obsessive–compulsive disorder (OCD), and autism spectrum disorder (ASD), are characterised by a pathological expression of such repetitive behaviours (RBs) in the form of tics, compulsions, or “stimming” behaviours, respectively.

Cortico-striatal circuits are the principal anatomical region that underlies the development of RB [4,5]. These circuits follow a parallel, mostly segregated, structural–functional organisation of limbic, associative, and sensorimotor pathways of information processing [6,7,8]. Different types of RBs are thought to differently recruit each loop [9]: motor tics, the main symptom of TS patients, have been described to be more related to alterations in the sensorimotor loop [10,11,12,13,14]. On the other hand, RBs consisting of a more complex sequence of actions or rituals, as is the case of compulsive-like behaviours, are frequently performed by OCD patients in response to intrusive thoughts or obsessions. The loops, related to these compulsive-like RBs, are the associative cortico-striatal circuits [15,16,17,18,19]. Despite this putative anatomical segregation of different types of RBs, a great percentage of comorbidity has been reported between TS and OCD patients, which points towards the existence of common anatomo-functional ground [20,21,22,23].

Rodent RBs have been extensively studied in translational psychiatric approaches, with a particular focus on excessive self-grooming as a behavioural marker for compulsive behaviours [24,25,26]. Self-grooming is an innate rodent behaviour that consists of a highly stereotyped sequence of rostro-caudal movements, essential for hygiene maintenance and well-being of the animal [24,25]. An aberrant manifestation of such a complex chain of movements is useful to study the mechanisms underlying the regulation and dysregulation of complex motor outputs such as compulsive-like behaviours. Currently, the reference mouse model for pathological RBs is the *Sapap3*-KO mouse [27,28,29,30]. These mice lack the synapse-associated protein 90/postsynaptic density protein 95 associated protein 3 (*Sapap3*), which is highly expressed in the cortex and striatum. The *Sapap3*-KO mouse model has been mainly reported and studied for its disproportionate and injurious levels of self-grooming, its increased anxiety, and its behavioural rescue with fluoxetine, a first-line treatment for OCD patients [27]. Consistent with clinical observations that the associative cortico-striatal loops are implicated in the emergence of compulsions, studies in this mouse model have shown a significant increase in the baseline firing rates of the medium spiny neurons (MSNs) in the major cortical input area of the associative circuits, the associative/centromedial striatum [28]. To further confirm the implication of the associative cortico-striatal circuits, we have previously shown that the optogenetic stimulation of the lOFC–centromedial striatal pathway restored self-grooming levels, likely through the recruitment of parvalbumin-positive (PV) interneurons [28]. Such implication of striatal PV interneurons has not only been observed in *Sapap3*-KO mice but also in other mouse models of pathological RBs [28,30,31,32]. Lastly, deficits in transsynaptic transmission in *Sapap3*-KO mice have been observed and shown to specifically occur in cortico-striatal but not thalamostriatal pathways, which also heavily project to the striatum [27,33]. More recently, a behavioural and pharmacological study of RBs in this mouse model has additionally detected tic-like RBs [34]. In rodents, tic-like behaviours, just as compulsive-like behaviours, rely on cortico-striatal circuits; however, in tic-like behaviours, sensorimotor circuits seem more strongly implicated [12,35].

Efficient connectivity and conductivity heavily depend on neuronal structure and integrity. On a population level, cell density and ratio can affect the integration and modulation of information; for example, in the context of RBs, a reduced number of PV interneurons has been shown to fail the regulation of striatal activity [12,28]. On a cellular level, the axonal structure can affect the speed of signal propagation [36]. Just minor alterations in the calibre of the axon, or the thickness of the myelin that insulates it, cause significant changes in conduction speed by altering the resistance to the conduction of the electrical signal [36,37]. The velocity of the signal conduction is, in its turn, determinant for either strengthening or weakening the connection between two synaptically connected neurons, thus being able to modulate neuroplasticity [38,39]. Therefore, it is rather intuitive that changes in these structural parameters have resurfaced as a potential neuroplasticity mechanism involved in learning and behaviour [40,41,42,43,44,45,46]. 

Different neuroimaging studies have found structural connectivity deficits in OCD patients [47,48]. However, given the limitations of non-invasive methods in humans, rodent models are required to advance our understanding of structural connectivity in the context of pathological RBs. Therefore, here, we raised the question of whether structural connectivity is affected in a comorbid model of tic- and compulsive-like RBs, using the *Sapap3*-KO mouse model. Hereby, we covered cortico-striatal candidate regions that have previously been characterised as affected both in human patients with tics or compulsions as well as in according animal models. We selected the associative (AS) and the sensorimotor (SMS) regions of the striatum and their respective major cortical projection areas, i.e., lOFC and primary/secondary motor cortex (M1/M2). In these candidate regions, we morphologically characterised myelinated axons in the *Sapap3*-KO mouse model, measuring both axon area and myelin thickness as the determinant parameters of axon conductivity. To achieve this, we took advantage of the open source software AxonDeepSeg [49], which automatically segments axon and myelin compartments using deep learning methods and performs various morphometric measurements (axon area, myelin thickness, g-ratio). AxonDeepSeg is fast (a few seconds per image), allowing for the analysis of hundreds of images in only a few minutes. As myelin coverage is performed by oligodendroglial cells, i.e., the glial cells that wrap around the fibres to produce the myelin sheaths, we additionally screened this cell type in the same regions of interest for potential abnormalities.

## 2. Materials and Methods

**Animals.** Adult *Sapap3*-KO and wild-type, age-matched controls (*Sapap3*-WT) (n = 32; aged 5–11 months) of C57BL/6J background were generated in heterozygous breeding trios. Animals were housed at the animal facilities of the Paris Brain Institute in Tecniplast, ventilated polycarbonate cages under positive pressure with hardwood bedding and provided with ad libitum food and water. The temperature was maintained at 21–23 °C and the relative humidity at 55 ± 10% with a 12 h light/dark cycle (lights on/off at 8 a.m. and 8 p.m., respectively). All experiments were approved by the French Ministry of Research under the agreement number (APAFIS) #1418-2015120217347265. Founders for the *Sapap3*-KO colony were kindly provided by Dr. Ann M Graybiel and Dr. G. Feng, MIT, Cambridge, USA. Genotyping was performed as in the original publication [27].

**Selection of regions of interest.** In order to determine the delimitations of the associative (AS) and sensorimotor striatum (SMS), we analysed anterograde projection patterns documented for the lateral orbitofrontal cortex (lOFC) and primary and secondary motor cortices (M1/M2) in the Allen Mouse Brain Connectivity Atlas [50]. Bregma levels resulting from this hodological screening were thus defined at +0.86 for the associative striatum (AS) and at +0.14 for the sensorimotor striatum (SMS) [51]. Bregma levels of the lOFC and M1/M2 areas corresponded to bregma = +2.68 and bregma = +1.94, respectively.

**Electron microscopy.** Animals (n = 4 per genotype) were deeply anaesthetized with pentobarbital (200 mg/kg) and transcardially perfused with fresh 5% glutaraldehyde (GA)/5 mM CaCl_2_. Brains were collected, post-fixed overnight in 5% GA at 4°, then rinsed and kept overnight in 0.12 M phosphate-buffered solution (PB) (pH = 7.4).

Brains were cut in 200 µm slices on the vibratome. Slices comprising the regions of interest were selected, and their left-hemispheric part punched under a stereomicroscope using a biopsy punch with a plunger 1 mm in diameter (PFM Medical). Punched samples were incubated with 4% osmium tetroxide in 100 mL sodium cacodylate buffer for one hour at RT and then carefully rinsed with sodium cacodylate buffer, three times. Next, samples were immersed in 5% uranyl acetate in 100 mL sodium cacodylate buffer for one hour and carefully rinsed in sodium cacodylate buffer, three times. The punches were then dehydrated in increasing concentrations of ethanol (50%, 70%, 90%, and twice 100%) for five minutes, and finally twice in 100% acetone for ten minutes. In order to prepare the samples for epoxy resin embedding, they were first immersed overnight, at 4 °C, in a solution of 50:50 acetone and epoxy resin. Next, samples were transferred into embedding moulds and immersed in pure resin for two hours. Finally, samples were left to polymerise at 60 °C for 48 h in a new dose of epoxy resin. Samples were then cut into 70 nm slices on an ultramicrotome, placed onto a Transmission Electron Microscope (TEM) grid and immersed in lead citrate for fifteen minutes. Samples were imaged at 100.0 k× for quantifications of axon calibre, myelin thickness and g-ratio, and at 6.2 k×, for the quantification of the density of myelinated axons in the Transmission Electron Microscope (HITACHI 120 kV HT7700, camera AMT XR41-B). In order to be specific on the quantification of local striatal cells, myelinated axons within the fibre bundles that cross the striatum, i.e., axons corresponding to the internal capsule, were excluded from analysis. 

**Electron microscopy analysis.***Myelin thickness and axon area/calibre measurements*. We quantified approximately 150 myelinated axons per region and animal using AxonDeepSeg [49]. In order to train the algorithm, we used a subset of TEM images and manually drew the inner and outer myelin layers using Fiji, ImageJ2. We then created an 8-bit image delineating the axon calibre area in white (i.e., area inside of the myelin inner layer), the myelin area in grey (i.e., area between the inner and the outer myelin layers), and the background area in black (i.e., area outside of the outer myelin layer). These ground truth masks were then provided to the algorithm together with the TEM images to train the model. After training, TEM images were fed into the trained model for automatic segmentation. At this step, we visually checked and corrected eventual artefacts before running the complementary algorithm that measured the area of the detected and segmented regions as inferred by pixel size. To validate the model, we manually analysed all AS images, confirming results as obtained through the algorithm. *Quantification of myelinated axons*. For each animal, we analysed 50 images per region of interest (AS, SMS, lOFC, M1/M2) and manually assessed the number of myelinated axons in each image, using Fiji, ImageJ2 [52,53]. In order to be specific on the quantification of local striatal cells, myelinated axons that form part of the internal capsule, i.e., fibre bundles that cross the striatum, were excluded (Figure 3a). 

**Immunohistochemistry.** Animals (n = 12 per genotype) were deeply anaesthetized with pentobarbital (200 mg/kg) and transcardially perfused with 2% PFA in 1X PBS. Fixed brains were dissected and post-fixed overnight in 2% PFA at 4 °C. Samples were immersed in 15% sucrose in 1X PBS for 24 h and subsequently immersed in 30% sucrose in 1X PBS for 48 h. Samples were then embedded in Tissue-Tek O.C.T. compound and frozen on dry ice. Brains were sliced coronally in 12 µm sections on a cryostat, transferred onto a coated glass slide (Superfrost Ultra Plus, Fisher Scientific, Illkirch, France), and stored at −80 °C. For immunohistochemical labelling, we performed heat-inducing antigen retrieval with an unmasking solution and, after cooling down at RT for twenty minutes, we rinsed the samples with 1X PBS three times for five minutes. Samples were then blocked in 4% BSA/0.1% Triton X-100 for 1 h. The primary antibodies against Oligodendrocyte transcription factor 2 (Olig2) mouse IgG2a, a nuclear marker for all cells of the oligodendroglial lineage, and against adenomatous polyposis coli clone CC1 (CC1) mouse IgG2b, a cytosolic marker specific for mature oligodendrocytes, were diluted in 4% BSA/0.1% Triton X-100 with a concentration of 1/500 and 1/100, respectively. The brain sections were incubated in this primary antibody solution overnight at 4°C. Samples were rinsed with 1X PBS and incubated in the dark with the secondary antibodies rat anti-Mouse IgG2a 488 and goat anti-Mouse IgG2b 555 Alexa in a 1/2000 dilution for 1 h at RT. The slides were rinsed under agitation in the dark, mounted in Mowiol mounting medium, coverslipped, and left overnight to dry at RT; hereby, the stained sections were protected from light. Fluorescent samples were imaged in a Zeiss Axio Observer 7 at 20× magnification. The ROIs were chosen visually by an experienced user and corresponded to one field of view (665.60 µm × 665.60 µm). We imaged both right and left hemispheres of two slices per region and animal, separated by 72 µm, summing up to a total of four images per region for each mouse.

**Immunohistochemistry analysis.** For the quantification of oligodendrocyte density, we used Fiji, ImageJ2 [52,53], and manually counted all cells that were positively stained for Olig2 and CC1. Positive immunolabelling for both Olig2 and CC1 (Olig2+/CC1+ cells) allowed for the identification of mature oligodendrocyte; positive immunolabelling of Olig2 but negative labelling of CC1 (Olig2+/CC1− cells) allowed for the identification of immature oligodendroglial cells. 

**Data analysis and statistics.** All data and statistical analysis were performed using Matlab R2017b. Given the small sample size, we exclusively performed non-parametric Mann–Whitney-U tests. We furthermore used two open source software packages available on GitHub for the visualisation of our data: raincloud plots [54] as well as estimation plots [55]. Cluster analysis was performed by fitting a Gaussian mixture model with two components (2 clusters) to our data [56]. 

## 3. Results

### 3.1. Axon Calibre Is Diminished in the Associative but Not in the Sensorimotor Striatum of Sapap3-KO Mice

**Figure 1:**
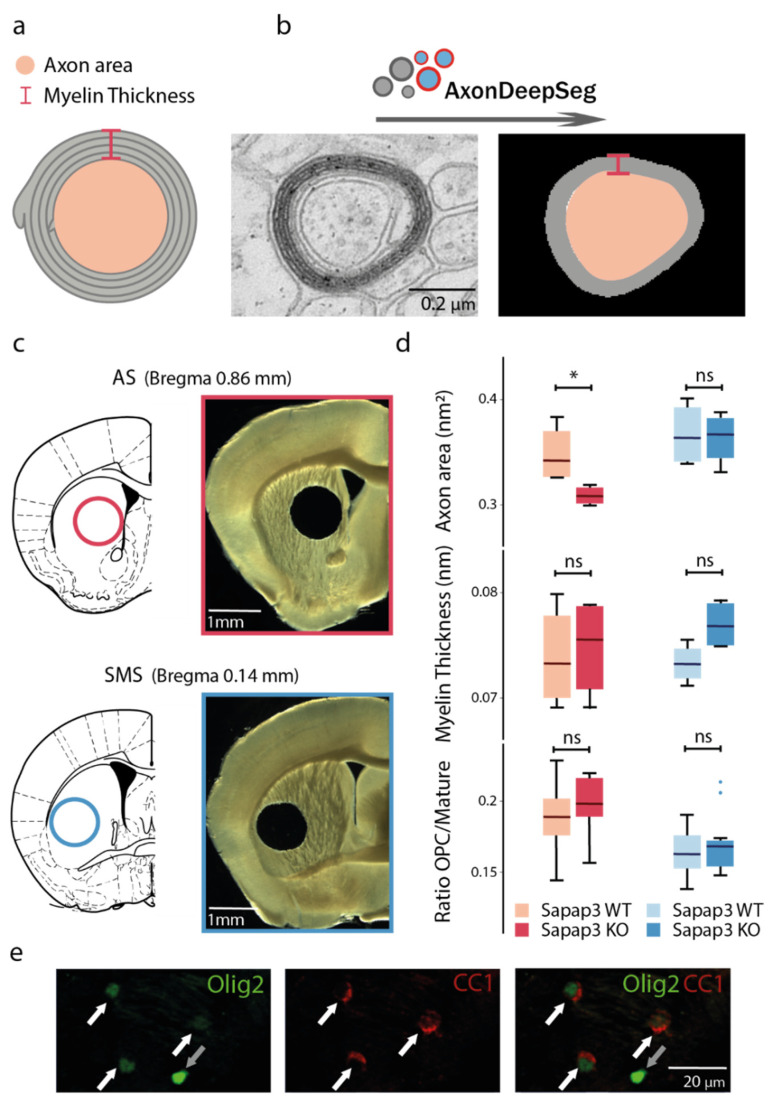
Axon calibre, not myelination, is altered in the associative striatum of the *Sapap3*-KO mouse. (**a**) Scheme of a myelinated axon highlighting the parameters of interest: the axon area (light pink) and myelin thickness (grey, delimitations in dark pink). (**b**) Schematic illustration of the proceeding by the AxonDeepSeg algorithm. Electron microscope images (100× acquisition) containing myelinated axons (left panel) are automatically detected, and measurements of axon surface and myelin thickness extracted (right panel). (**c**) Schemes (left panels) and stereomicroscopic images (right panels) of coronal brain slices at target bregma levels illustrating punch-extraction of the regions of interest of the associative (red outlines) and the sensorimotor striatum (blue outlines). (**d**) Axon area, myelin thickness, and ratio between immature and mature oligodendroglial cells in *Sapap3*-KO (darker colours) and wild-type controls (lighter colours) in the associative (red shades) and the sensorimotor striatum (blue shades). Box-whisker plots illustrate twenty-fifth and seventy-fifth percentiles, respectively, and medians. *: *p* < 0.05; ns = non-significant. (**e**) Exemplary immunofluorescence image (20× magnification) of immature (Olig2+/CC1−; grey arrows) and mature (Olig2+/CC1+; white arrows) oligodendrocytes, with split (left and middle panel) and merged channels (right panel). Olig2 signal is pseudo-coloured in green, CC1 signal in red. AS = associative striatum; SMS = sensorimotor striatum.

In order to assess the striatal structural integrity of the *Sapap3*-KO mouse model of tic- and compulsive-like behaviours, we characterised axon calibre and myelin thickness, two parameters that crucially contribute to axonal conductivity (Figure 1a). Taking advantage of a deep learning algorithm, AxonDeepSeg, which is capable of automatically, rapidly, and accurately segmenting and measuring myelinated axons from electron microscopy data in an unbiased manner [49] (Figure 1b), we assessed the properties of approximately 150 axons per region and animal. Applying such a tool, we measured axon area and myelin thickness, i.e., the difference between the outer and inner myelin layer (Figure 1b). The latter was extracted via the corresponding outer and inner axonal surfaces. Hereby, we distinguished between associative and sensorimotor striatal regions, i.e., striatal areas receiving projections from the respective cortical areas [50]. These are located at the medial or lateral striatal boundary along the dorso-central continuum, respectively (Figure 1c).

We found that axon area was significantly diminished in the associative striatum of the *Sapap3*-KO mice in comparison with their wild-type, age-matched controls (median_WT_ = 0.34 vs. median_KO_ = 0.31; Mann–Whitney U: U = 16, *p*-value = 0.03). However, there was no difference in axon area in the sensorimotor striatum (median_WT_ = 0.36 vs. median_KO_ = 0.37; Mann–Whitney U: U = 9, *p*-value = 0.89) (Figure 1d, upper panels). On the other hand, myelin thickness in *Sapap3*-KO mice was unaltered in either striatal region (AS: median_WT_ = 0.07 vs. median_KO_ = 0.08; Mann–Whitney U: U = 8, *p*-value = 1; SMS: median_WT_ = 0.07 vs. median_KO_ = 0.08; Mann–Whitney U: U = 2 *p*-value = 0.11) (Figure 1d, centre panels). Additionally, we calculated the g-ratio, a classical measurement that describes the myelin thickness in proportion to the calibre of the axon by dividing the radius of the inner layer by the radius of the outer layer. No difference between *Sapap3*-KO and control mice was detected in g-ratio measurements either in the associative (n = 4 per genotype; median_WT_ = 0.80 vs. median_KO_ = 0.79; Mann–Whitney U: U = 12, *p*-value = 0.34) or the sensorimotor striatum (n = 4 per genotype; median_WT_ = 0.81 vs. median_KO_ = 0.80; Mann–Whitney U: U = 13, *p*-value = 0.20; Supplementary Figure S1, upper panels).

These results suggested that myelination per se might not be affected in *Sapap3*-KO mice. However, in order to utterly exclude potential changes on myelination itself, we furthermore histologically analysed the cells from the oligodendroglial lineage, i.e., those cells which wrap around and myelinate the fibres. Thus, we performed immunohistological stainings in order to quantify the overall population of oligodendroglial cells, using oligodendrocyte transcription factor 2 (Olig2) as a cell marker. For the pool of mature oligodendrocytes, i.e., those cells which actively perform myelination, we applied the criterion of a positive double-immunolabelling for both Olig2 as well as adenomatous polyposis coli clone CC1 (CC1) (Figure 1e, white arrows). Cells that were only Olig2-positive but CC1-negative corresponded to immature oligodendroglial cells (Figure 1e, grey arrows). In line with our AxonDeepSeg results of unaltered myelin thickness in the associative and sensorimotor striatum of *Sapap3*-KO mice, no significant differences were observed either in the number of immature (n = 12 per genotype; AS: median_WT_ = 91.56 vs. median_KO_ = 94.38; Mann–Whitney U: U value = 60, *p*-value = 0.51; SMS: median_WT_ = 75.90 vs. median_KO_ = 71.95; Mann–Whitney U: U = 76.5, *p*-value = 0.82; Supplementary Figure S1, centre panels) or in the number of mature, i.e., myelinating oligodendrocytes (n = 12 per genotype; AS: median_WT_ = 517.18 vs. median_KO_ = 497.72; Mann–Whitney U: U = 80, *p*-value = 0.67; SMS: median_WT_ = 441.85 vs. median_KO_ = 445.24; Mann–Whitney U: U = 77, *p*-value = 0.80; Supplementary Figure S1, bottom panels). We reasoned that maybe a different proportion of mature oligodendrocytes would give insight into the eventual maturation deficits of this cell line. Therefore, we additionally assessed the ratio of immature versus mature oligodendrocytes, yet did not detect significant differences (n = 12 per genotype; AS: median_WT_ = 0.19 vs. median_KO_ = 0.20; Mann–Whitney U: U = 115, *p*-value = 0.16; SMS: median_WT_ = 0.16 vs. median_KO_ = 0.17; Mann–Whitney U: U = 131, *p*-value = 0.62) (Figure 1d, bottom panels). 

The striatum is the main input region of cortical projections, and *Sapap3* deletion has been shown to specifically affect cortico-striatal but not, for example, thalamostriatal projections [33]. Hence, we next assessed whether the detected reduction of axon area was specific to the associative striatum or whether it was a feature of overall cortico-striatal circuitry. Therefore, we expanded the analysis of axon area and myelin thickness to the lateral OFC (lOFC) as well as to the primary and secondary motor cortex (M1/M2), i.e., those cortical regions that provide respective major cortical inputs into the associative and sensorimotor striatum (Supplementary Figure S2a). We found no significant difference in either parameters in lOFC (n = 4 per genotype; axon area: median_WT_ = 0.46 vs. median_KO_ = 0.46; Mann–Whitney U: U = 8, *p*-value = 1; Supplementary Figure S2b, upper panels; myelin thickness: median_WT_ = 0.08 vs. median_KO_ = 0.08; Mann–Whitney U: U = 5, *p*-value = 0.49; Supplementary Figure S2b, centre panels) as well as M1/M2 (n = 4 per genotype; axon area: median_WT_ = 0.55 vs. median_KO_ = 0.50; Mann–Whitney U: U = 13, *p*-value = 0.20; Supplementary Figure S2b, upper panels; myelin thickness: median_WT_ = 0.08 vs. median_KO_ = 0.08; Mann–Whitney U: U = 7, *p*-value = 0.89; Supplementary Figure S2b, centre panels). In the same line, we did not find any differences in the ratio of oligodendroglial cells (n = 12 per genotype; lOFC: median_WT_ = 0.27 vs. median_KO_ = 0.30; Mann–Whitney U: U = 63, *p*-value = 0.62; M1/M2: median_WT_ = 0.31 vs. median_KO_ = 0.30; Mann–Whitney U: U = 72, *p*-value = 1; Supplementary Figure S2b, bottom panels).

### 3.2. The Reduction in Axon Calibre Arises from a Subpopulation of Axons

**Figure 2:**
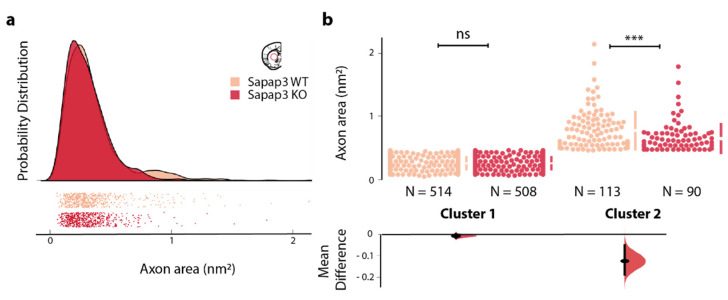
Axon area difference in the associative striatum of *Sapap3*-KO mice originates from a subpopulation of neurons. (**a**) Individual measurement of axon area in the associative striatum of *Sapap3*-KO and wild-type (WT) mice (below) and raincloud plot of their probability distribution (on top). Separate peaks in the probability distribution suggest the presence of two clusters in both *Sapap3*-KO and wild-type mice. (**b**) Axon areas of myelinated axons (individual data points; upper panel) form two clusters (cluster 1, left; cluster 2, right) in the associative striatum of *Sapap3*-KO and wild-type mice (colour coding as above) as confirmed by fitting a two-component Gaussian mixture model. The axon area of myelinated axons in cluster 2 is significantly reduced in *Sapap3*-KO compared to wild-type mice (lower panel). *Sapap3*-KO mice are depicted in dark, wild-type mice in light red. ***: *p* < 0.001; ns = non-significant.

Having detected a significant decrease in the axon area of the *Sapap3*-KO mice in the associative striatum, we pondered whether this reduction would arise from an overall reduced calibre of myelinated axons or rather from a reduction specific to a particular range of axon populations. As a third option, we furthermore considered the possibility of a reduction in the number of a specific subpopulation of axons characterised by a large calibre, hereby skewing the overall calibre size to a smaller average in the *Sapap3*-KO mice. In order to investigate these three different possibilities, we plotted the probability distribution of all axon areas, segregating *Sapap3*-KO and *Sapap3*-WT groups, using the Raincloud plot algorithm [54]. The obtained probability density plot suggested a bimodal distribution of axon area of *Sapap3*-WT mice; this distribution appeared blunted in the *Sapap3*-KO (Figure 2a). Attending to the considerable heterogeneity of striatal cells and the observed bimodal distribution, we fitted a two-component Gaussian mixture model to our data in order to test for the potential presence of two different clusters. Indeed, this model confirmed the visual impression of a bimodal distribution and detected the presence of two clusters: the first, larger cluster contained 85% of axons of the *Sapap3*-KO group, and 82% of the *Sapap3*-WT group (Cluster 1), and the other cluster contained 15% and 18% of axons (Cluster 2), respectively (Figure 2b).

We verified between-subject comparability of the distributional profiles (Supplementary Figure S3). Axon areas in Cluster 1, which comprises the vast majority of the axons, were similar between *Sapap3*-KO and *Sapap3*-WT mice (n_WT_ = 514, n_KO_ = 508; median_WT_ = 0.26 vs. median_KO_ = 0.24; Mann–Whitney U: U = 131604.5, *p*-value = 0.29). However, axons of *Sapap3*-KO mice assigned to Cluster 2 were significantly smaller than those of their wild-type, age-matched controls (n_WT_ = 113, n_KO_ = 90; median_WT_ = 0.67 vs. median_KO_ = 0.58; Mann–Whitney U: U = 7986.0, *p*-value = 6.52 × 10^−4^) (Figure 2b).

These results suggest that the observed reduction in axon calibre in the associative striatum of *Sapap3*-KO mice is specific to a small subpopulation of axons characterised by larger axons, here assigned to Cluster 2. Thus, reduced axon calibre is not a general feature of all myelinated axons in the associative striatum.

**Figure 3:**
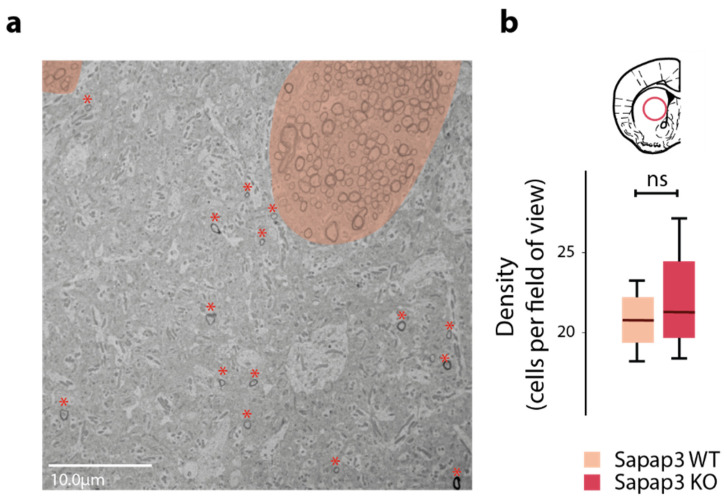
Density of myelinated axons is not altered in the associative striatum of *Sapap3*-KO mice. (**a**) Exemplary electron microscopy image (6.2 k× magnification). The internal capsule, which was excluded from analysis, is highlighted in light pink. Quantified axons beyond the internal capsule are indicated by dark pink asterisks. (**b**) Density of myelinated axons in the associative striatum of *Sapap3*-KO (dark red) and wild-type mice (light red). Box-whisker plots illustrate the twenty-fifth and seventy-fifth percentiles and the median. ns = non-significant.

However, one further hypothesis remained to be explored: was the genotype difference in Cluster 2 due to an overall reduction of axon calibre within that cluster? Or could this difference be explained by the relative lack of a subpopulation of large axons within Cluster 2, hereby skewing axon calibre averages within that cluster to a smaller average in *Sapap3*-KO mice? The latter possibility would be reflected in a decreased overall number of myelinated axons in the associative striatum of *Sapap3*-KO mice. Hence, we quantified and averaged the density of myelinated axons in 50 transmission electron microscopy images per animal in the associative striatum, again comparing *Sapap3*-KO to age-matched, wild-type controls. Attending to the fact that the striatum is traversed by innumerous fibres of the internal capsule, which largely project outside of the striatum, we visually excluded the bundles of the internal capsule (Figure 3a). We did not detect any difference in the density of myelinated axons in the associative striatum between *Sapap3*-KO and *Sapap3*-WT animals (n = 4 per genotype; median_WT_ = 20.80 vs; median_KO_ = 21.41; Mann–Whitney U: U = 6, *p*-value = 0.69; Figure 3b). This result suggests that a lack of a subgroup of axons with large calibre cannot account for the axon calibre difference in the associative striatum of *Sapap3*-KO mice. Thus, the observed genotype-dependent difference in axon calibre in the associative striatum is likely due to an overall reduction in axon calibre of Cluster 2.

## 4. Discussion

In the present study, we demonstrated that structural connectivity is affected in the *Sapap3*-KO mice, a mouse model of comorbid compulsive- and tic-like behaviours [34]. This deviation consists of a reduced axon calibre of myelinated fibres, specifically in the associative striatum. Such a structural deficit was not detected either in the sensorimotor striatum or in upstream cortical regions, namely, lOFC and M1/M2, respectively. Furthermore, we provide evidence that this reduction in axon calibre affects only a small cluster of axons characterised by a large calibre, which represent less than 20% of the overall number of myelinated axons in the associative striatum. Myelination itself, however, as assessed through myelin thickness and histological quantification of the myelin-generating oligodendrocytes, seems unaffected. Even though myelin thickness is dependent on the diameter of the axon [57], it has been shown that diameter is not the only parameter for myelin regulation [58]; i.e., axon calibre could thus be altered independently of changes in myelin thickness.

Axon calibre is the main regulator of conduction speed [59,60]. In its turn, a diminished conduction speed affects synaptic transmission and thus transsynaptic communication with downstream cells [38,39]. Indeed, it has been demonstrated in primate motor callosal neurons that a smaller axon diameter corresponds to smaller synaptic bouton size and, consequently, smaller neurotransmission release [61]. In general, structural parameters have been shown to contribute to deficient neuronal signal propagation in circuits affected by major psychiatric disorders such as schizophrenia or ASD: hypomyelination specific to cortical PV interneurons has been reported in a rat model of schizophrenia [62]. Additionally, patients with ASD, a developmental behavioural disorder frequently characterised by the presence of RBs in the form of “stimming” behaviours, display a smaller axon calibre in the myelinated axons of the corpus callosum when compared to healthy controls [63]. Hence, the reduced axon calibre in the associative striatum of *Sapap3*-KO mice further corroborates a connectivity deficit in associative cortico-striatal circuits as previously suggested by studies using in vivo and vitro electrophysiology [28,31,33,64]. Our results are thus in line with both the large fraction of the clinical literature on OCD reporting abnormalities in cortico-striatal associative circuits [15,16,18,19,65,66], as well as the research focused on compulsive-like behaviours and associative cortico-striatal circuits in the *Sapap3*-KO mouse model. However, while our results are in resonance with the focus on associative cortico-striatal circuits, sensorimotor cortico-striatal macrocircuitry has emerged as a relevant candidate for neuropsychiatric disorders with pathological RBs. For several years, the clinical literature has pronounced the heterogeneity of OCD and important comorbidity between tic-like and compulsive-like behaviours in both TS and OCD patients [20,21,22,23]. This is highly relevant for the *Sapap3*-KO mouse model, given the recent detection of tic-like movements in addition to compulsive-like grooming behaviours in this mouse model [34]. In a mouse model that spans such a complex spectrum of tic- and compulsive-like RBs, we were surprised to find that axon calibre was affected only in the associative striatum, while the sensorimotor striatum was found to be unaltered. How could altered axon calibre specific to the associative striatum explain such a comorbid behavioural phenotype, for which, in the most straightforward manner, both an associative as well as a sensorimotor circuitry component could be expected [10,11,12,15,16,17,18,19]? One possible explanation for the presence of tic-like movements in this mouse model could be due to a strong sensorimotor cortical input from M2 into the associative striatum in addition to that from associative cortical areas [30]. Indeed, Corbit et al. described a substantially increased input from M2 onto both striatal MSNs and parvalbumin-positive (PV) interneurons in the centromedial striatum in *Sapap3*-KO mice. This suggests a maladaptive reinforcement of sensorimotor relative to associative areas, which could lead to aberrantly elevated motor output. The implication of both associative as well as sensorimotor cortico-striatal loops is further supported by the finding of synaptic alterations in both circuits in young adult *Sapap3*-KO mice: cingulate area 1/M2 cortical projections to the dorsomedial striatum (DMS) and primary motor/M2 cortical projections to the dorsolateral striatum (DLS) [64]. A concomitant decrease in associative circuits, as suggested in our study, could further corroborate such an imbalance between sensorimotor and associative circuits, which has also been previously suggested for patients and other animal models of RBs [67,68]. One possible scenario might be that such an imbalance could, for example, facilitate the disruption of the processing of complex and sequential motor outputs in favour of breaking them into simpler, shorter, and purely motor behaviours.

Currently, a knowledge gap on myelination of specific cell types, including in the striatum, hampers the deduction of the cell identity from the size of the axon calibre of cluster number 2. Indeed, the present-day state of knowledge does not allow for the conclusion as to whether the observed structural striatal deviation arises from afferents such as cortico-striatal or thalamostriatal connections or whether the reduced axon calibre represents an inherent striatal microcircuitry dysfunction. For example, to date, there is no direct evidence whether cortico-striatal pathways are actually myelinated [69,70,71] or not [72,73]. Knowledge about myelination of striatal microcircuitry remains equally limited. Myelination has been demonstrated for certain striatal cell types, such as MSNs [74], PV [75], or ChAT [76] interneurons, but not, for example, dopaminergic striatal afferents [77,78]. Nevertheless, the proportion of myelinated axons of each cell type and the proportion of myelinated versus unmyelinated collaterals [79,80,81] is variable and yet remains to be investigated. Additionally, potential cell-type-specific parameters such as myelin thickness spectra or axon calibre range require further fundamental investigation. Hopefully, nowadays, available neuroanatomical tracing and imaging techniques will serve to unravel these important neuroanatomical structural features in the near future. Taken together, the current lack of knowledge on cell-type-specific myelination does not allow for the identification of cells in Cluster 2 by means of assessed axon calibre ranges, proportions of myelinated axons, or myelin thickness. Nevertheless, three cell-type-specific candidates for pathological RBs in patients [82,83] and rodent models [28] derserve a closer structural characterisation in the future: cortico-striatal projections, as well as PV- or ChAT-positive striatal interneurons.

In the first scenario, reduced axon calibre in the associative striatum could be explained as a macrocircuitry dysfunction of cortico-striatal loops. Concretely, the observed reduction in axon calibre in a small cluster of striatal axons might arise from a population of associative cortico-striatal projection neurons. Given that axonal calibre is positively related to synaptic strength [38,39], the here-observed structural phenotype of a decrease of axon calibre in the associative striatum is in line with and corroborates a previously reported decrease in cortico-striatal synaptic strength in the associative striatum [30,64]. Cortico-striatal tracing studies will be necessary in the future to further investigate this first structural macro-circuitry hypothesis.

In a second scenario, Cluster 2 neurons might correspond to PV-positive interneurons. These interneurons are crucially implicated in regulating striatal activity through a powerful mechanism of fast-forward inhibition [84,85], and they are reduced in number in the associative/centromedial striatum of the *Sapap3*-KO mouse [28]. Consequently, in the same study, we hypothesised that optogenetic stimulation of the lOFC leads to the recruitment of PV interneurons, which might have a role in reducing compulsive-like behaviours [28]. A reduced axonal calibre arising specifically from the subpopulation of PV interneurons would be consistent with previously observed diminished cortico-striatal synaptic activity in general [61] and with decreased numbers of PV interneurons specifically [28,64]. Altered striatal micro-circuitry in the form of a weakened striatal inhibitory PV interneuronal network has been described both in patients with aberrant repetitive behaviours [82,83] as well as in the striatum of several mouse models [31,86], including in the associative striatum of *Sapap3*-KO mice [28,64]. PV interneurons, even though representing a small percentage of the total number of striatal cells, form a strong inhibitory blanket across the striatum. Alterations in these cells, either by a reduction in size or through a deficient morphology and connectivity with downstream cells, reduce their feed-forward inhibitory power, failing, therefore, to finely orchestrate the MSNs in their role of behavioural control [28,87].

In a third scenario, striatal interneurons expressing choline acetyltransferase (ChAT) also need to be considered as a candidate for Cluster 2 neurons. ChAT-positive interneurons, though sparse, highly contribute to the modulation of MSNs [88]. ChAT-positive interneurons have been described as reduced in number in TS patients [83], and their partial ablation produces tic-like behaviours in mice [12], as well as compulsive social behaviours [89]. 

In summary, our study corroborates previous findings of deficits specifically within the associative striatum of the *Sapap3*-KO mouse, here assessed for the first time using a structural, deep learning-based approach. This study thus contributes a novel and complementary aspect to the discussion of deviations in macro- and microcircuitry in the context of RBs in clinical as well as fundamental studies. Future structural neuroanatomy studies are required to define the affected striatal cell type within the pool of currently prominent candidates, such as cortico-striatal projections or PV- or ChAT-positive striatal interneurons.

## 5. Conclusions

In our study, we found that axon calibre in the associative striatum is reduced in the *Sapap3*-KO mouse. This axon caliber reduction was not found in cortical areas projecting onto the associative striatum and neither in sensorimotor cortico-striatal regions. The difference in axon calibre arises from a smaller cluster of cells, the identity of which will need to be determined in neuroanatomic follow-up studies. Potential cell-type specific candidates for this cluster, in the context of pathological RB, include cortico-striatal projections, PV- or ChAT-positive striatal interneurons. Our results further corroborate, from a structural point of view, neurophysiological deficits previously observed in associative cortico-striatal circuits of the *Sapap3*-KO mouse, which are known to be implicated in compulsive-like behaviours. 

## Data Availability

The datasets and analyses of this study are available from the corresponding author on reasonable request.

## Supplementary Materials

The following are available online at https://www.mdpi.com/article/10.3390/brainsci11101353/s1, Figure S1: G-ratio and oligodendroglial cells are not altered in the striatum of the *Sapap3*-KO mouse. Figure S2: Axon area, myelin thickness, and oligodendroglial cells are not altered in the cortical input regions of the *Sapap3*-KO mouse. Figure S3: Individual subject probability distribution of axon area.
