# Supplementary material for: Reduced Axon Calibre in the Associative Striatum of the Sapap3 Knockout Mouse
Source: Brain Sci. 2021 Oct 14;11(10):1353. doi: 10.3390/brainsci11101353 (PMC8570333; doi:10.3390/brainsci11101353)
Supplement: Supplementary file 1 [file brainsci-11-01353-s001.zip › BrainSciences_Lousada_et_al_Supplement.pdf]

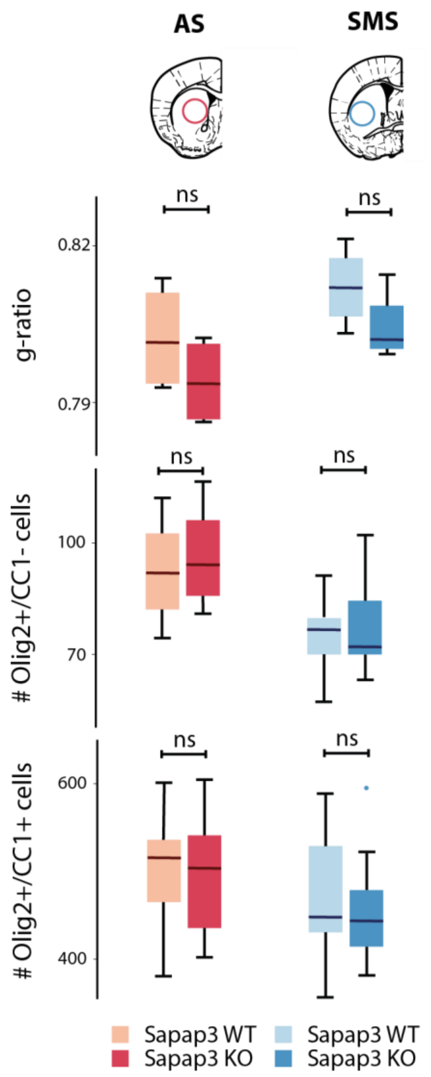


**Supplementary Figure S1: G-ratio and oligodendroglial cells are not altered in the striatum of the *Sapap3*-KO mouse.** G-ratio (upper panel), number of Olig2+/CC1-, i.e. immature oligodendroglial cells (center panel), and number of Olig2+/CC1+ cells, i.e. mature oligodendrocytes (bottom panel) in *Sapap3*-KO (darker colors) and wild-type controls (lighter colors) in the associative (red shades) and the sensorimotor striatum (blue shades). Box-Whisker plots illustrate 25th and 75th percentiles respectively, and medians. ns = non-significant.

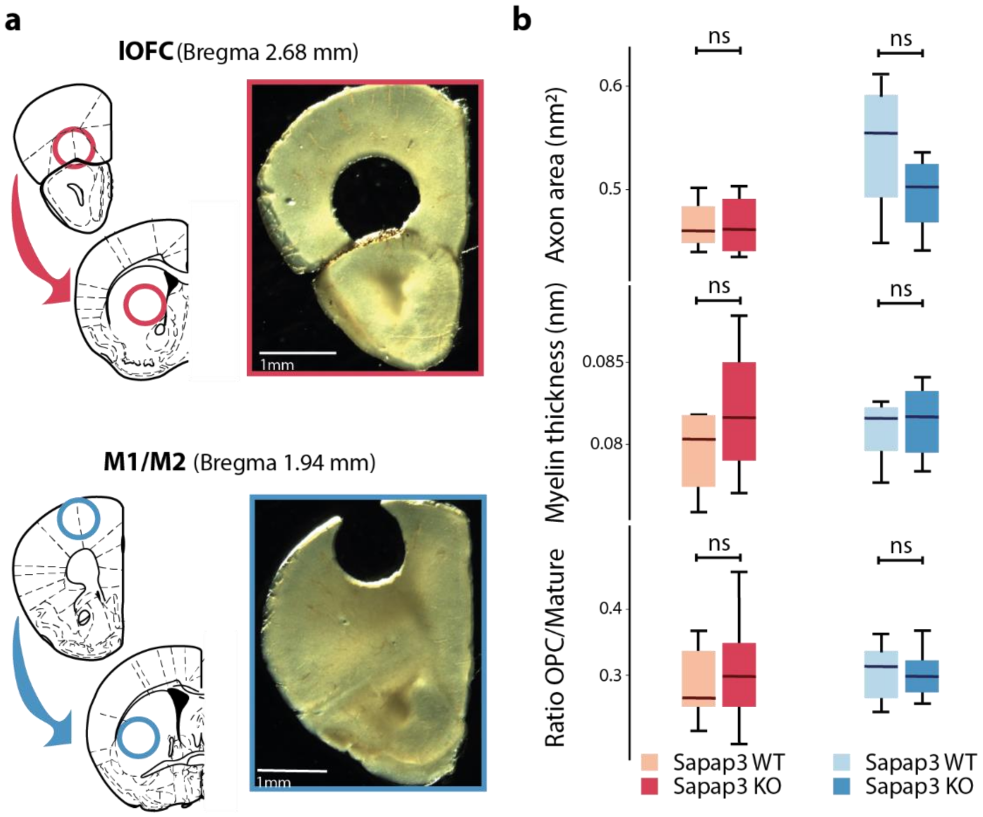


**Supplementary Figure S2. Axon area, myelin thickness, and oligodendroglial cells are not altered in the cortical input regions of the Sapap3-KO mouse.** (**a**) Schemes (left panels) and stereomicroscopic images (right panels) of coronal brain slices at target bregma levels illustrating punch-extraction of the regions of interest, which provide important input to the associative or sensorimotor striatum, i.e. lateral OFC (red outlines) and the M1/M2, respectively (blue outlines). (**b**) Axon area, myelin thickness, and ratio between immature and mature oligodendroglial cells (upper, center, and bottom panels, respectively) in *Sapap3*-KO (darker colors) and wild-type controls (lighter colors) in the OFC (red shades) and the M1/M2 (blue shades). Box-Whisker plots illustrate 25$^{th}$ and 75$^{th}$ percentiles respectively, and medians. ns = non-significant.

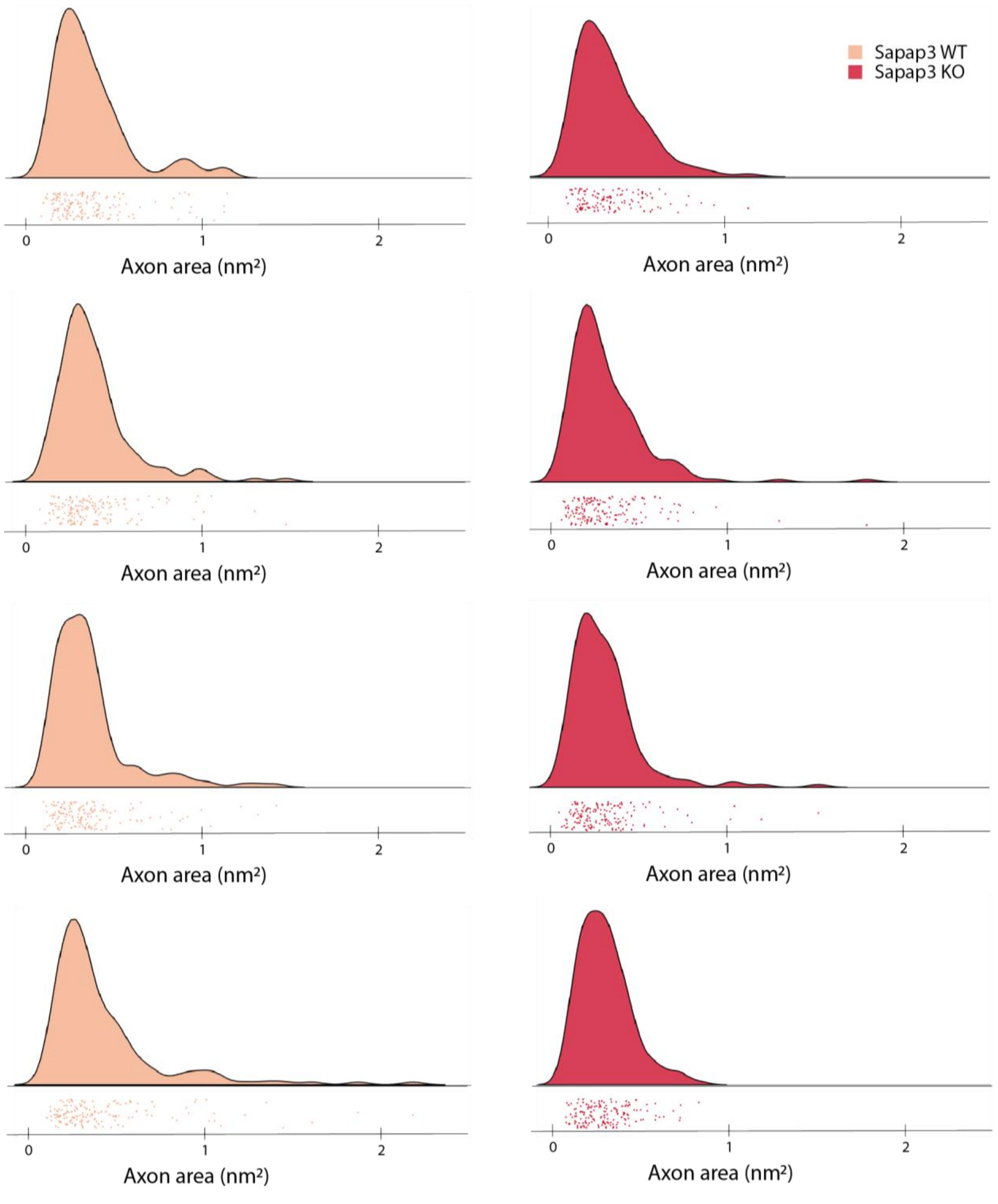


**Supplementary Figure S3. Individual subject probability distribution of axon area.** Individual axon area data points of the associative striatum of *Sapap3*-KO (dark pink) and wild-type mice (light pink) (below each individual plot) and raincloud plot of their probability distribution (top of each individual plot).
